# The “hefty hamate hook” sign of pisiform-hamate coalition

**DOI:** 10.1007/s00247-025-06220-7

**Published:** 2025-03-22

**Authors:** Madalyn Snoddy, Lisa Betz

**Affiliations:** Department of Radiology and Radiological Science, Medical University of South Carolina, 96 Jonathan Lucas Street, Suite 210, MSC 323, Charleston, SC 29425 USA

**Keywords:** Carpal coalition, Pisiform, Hamate


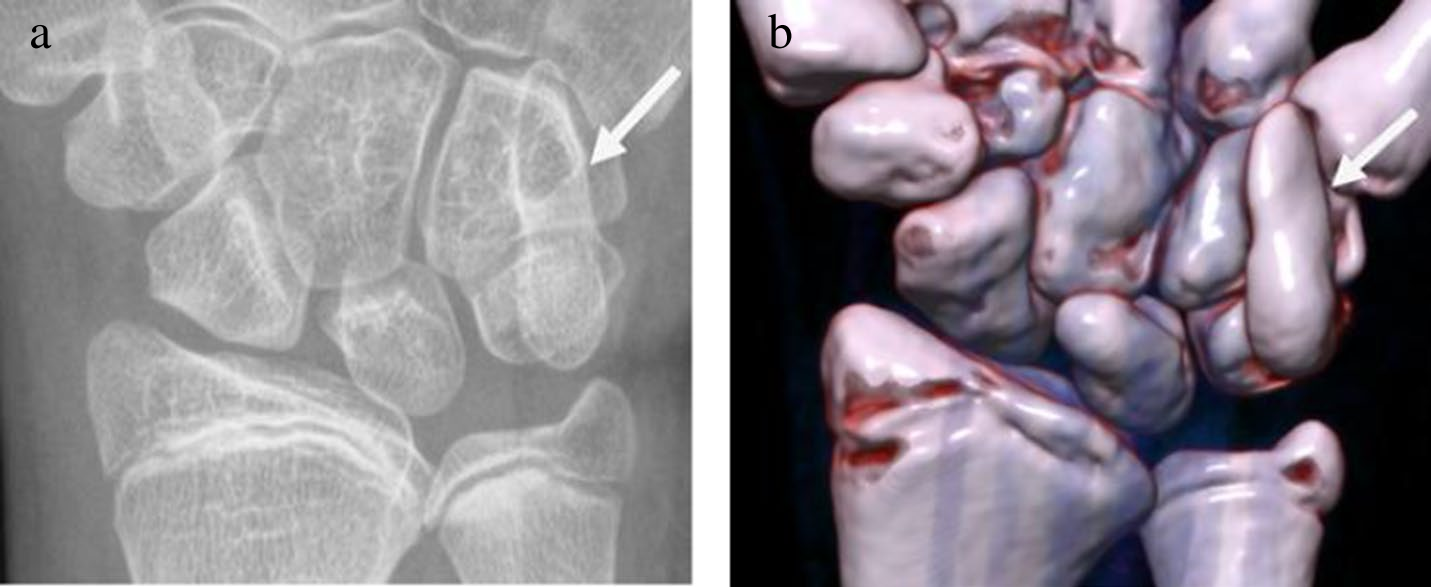


A 16-year-old female with Lennox-Gastaut syndrome was evaluated for asymptomatic right wrist swelling. PA radiograph (**a**) revealed an enlarged hook of the hamate (arrow). 3D reconstructions of a CT (**b**) confirmed osseous pisiform-hamate coalition (*arrow*). The hook of the hamate (also known as the hamulus) and pisiform bone form a distinctive connection in osseous pisiform-hamate coalition. The appearance of a “hefty hamate hook” has not been reported previously but may be a sign to help diagnose this condition on radiographs. Pisiform-hamate coalition is uncommon and differs from other wrist coalitions by involving both carpal rows. To our knowledge, it has not been associated with Lennox-Gastaut syndrome. Although this coalition is often asymptomatic, it is more prone to fracture and can be a source of ulnar nerve entrapment.

## Data Availability

No datasets were generated or analysed during the current study.

